# Supplementary material for: The structure of the core NuRD repression complex provides insights into its interaction with chromatin
Source: eLife. 2016 Apr 21;5:e13941. doi: 10.7554/eLife.13941 (PMC4841774; doi:10.7554/eLife.13941)
Supplement: Figure 3—source data 1. — Intermolecular crosslinks are listed with the respective proteins and amino acids identified. Crosslinks with an xQuest score greater than 14.5 are included. Crosslinks that fit the model are numbered. Crosslinks that do not fit the model are indicated "x" and we presume result from low levels of aggregation between complexes. DOI: http://dx.doi.org/10.7554/eLife.13941.009 [file elife-13941-fig3-data1.docx]

**Figure 3 – source data 1.**

**Crosslinks within the MTA1-B (162-546):HDAC1:RBBP4 complex.**

**__________________________________________________________________**

Protein1 Protein2 nAA1 nAA2 xQuest Consistent with SAXS

Score model?

MTA1 HDAC1 164 74 16.83 1

MTA1 HDAC1 164 218 14.84 2

MTA1 HDAC1 340 279 14.72 3

MTA1 HDAC1 343 50 22.85 4

MTA1 HDAC1 343 363 21.09 5

MTA1 HDAC1 350 50 17.25 6

MTA1 HDAC1 350 58 15.31 x

MTA1 HDAC1 426 218 15.12 x

MTA1 HDAC1 426 283 24.34 7

MTA1 HDAC1 426 289 24.93 8

MTA1 HDAC1 462 74 16.08 x

MTA1 HDAC1 462 218 18.35 9

MTA1 HDAC1 462 363 24.43 10

MTA1 HDAC1 466 126 16.46 x

MTA1 HDAC1 466 218 19.25 11

MTA1 HDAC1 466 279 20.28 12

MTA1 HDAC1 477 218 15.91 x

MTA1 HDAC1 532 89 20.45 x

MTA1 HDAC1 532 218 17.31 x

MTA1 RBBP4 164 156 18.76 13

MTA1 RBBP4 340 22 15.03 x

MTA1 RBBP4 340 120 16.11 14

MTA1 RBBP4 343 160 17.11 15

MTA1 RBBP4 350 120 21.34 16

MTA1 RBBP4 426 22 14.87 17

MTA1 RBBP4 426 160 23.17 18

MTA1 RBBP4 426 309 18.45 x

MTA1 RBBP4 431 307 18.83 x

MTA1 RBBP4 462 160 17.35 19

MTA1 RBBP4 466 114 14.98 20

MTA1 RBBP4 466 307 21.16 x

MTA1 RBBP4 466 309 18.17 x

MTA1 RBBP4 532 309 16.1 21

HDAC1 RBBP4 218 4 22.9 22

HDAC1 RBBP4 218 22 19.97 23

HDAC1 RBBP4 283 215 14.73 24

HDAC1 RBBP4 363 120 14.61 25
