# Supplementary material for: The structure of the core NuRD repression complex provides insights into its interaction with chromatin
Source: eLife. 2016 Apr 21;5:e13941. doi: 10.7554/eLife.13941 (PMC4841774; doi:10.7554/eLife.13941)
Supplement: Figure 6—source data 1. — The key includes the identity and post-translational modification state of the histone peptides in the arrays presented in Figure 6a and Figure 6—figure supplement 1. The strength of observed binding is approximated by one or two asterisks. DOI: http://dx.doi.org/10.7554/eLife.13941.017 [file elife-13941-fig6-data1.docx]

**Figure 6 – source data 1.**

**Key for the MODified histone peptide array – Rows J1 to P24**

* Indicates peptide binding; ** indicates intense binding observed

**Peptide MTA1:**

**location RBBP4 RBBP4 name Mod1 Mod2 Mod 3 Mod 4 N-terminus**

J 1 H3 1-19 R2me2a K4me2 R8me2a K9ac free

J 2 H3 1-19 R2me2a K4me3 R8me2a K9ac free

J 3 H3 1-19 R2me2a K4ac R8me2a K9ac free

J 4 H3 7-26 unmod acetylated

J 5 H3 7-26 K14ac acetylated

J 6 H3 7-26 K14ac S10P acetylated

J 7 H3 7-26 K14ac T11P acetylated

J 8 H3 7-26 R17me2s acetylated

J 9 H3 7-26 R17me2a acetylated

J10 H3 7-26 R17Citr acetylated

J11 H3 7-26 K18ac acetylated

J12 H3 7-26 K14ac R17me2s acetylated

J13 H3 7-26 K14ac R17me2a acetylated

J14 H3 7-26 K14ac K18ac acetylated

J15 H3 7-26 R17me2s K18ac acetylated

J16 H3 7-26 R17me2a K18ac acetylated

J17 H3 7-26 R17Citr K18ac acetylated

J18 H3 7-26 K14ac R17me2s K18ac acetylated

J19 H3 7-26 K14ac R17me2a K18ac acetylated

J20 H3 16-35 unmod acetylated

J21 * H3 16-35 R26me2s acetylated

J22 * H3 16-35 R26me2a acetylated

**J23 ** ** H3 16-35 R26Citr acetylated**

J24 H3 16-35 K27me1 acetylated

K 1 * H3 16-35 K27me2 acetylated

K 2 * H3 16-35 K27me3 acetylated

**K 3 ** ** H3 16-35 K27ac acetylated**

K 4 H3 16-35 S28P acetylated

K 5 H3 16-35 R26me2s K27me1 acetylated

K 6 * * H3 16-35 R26me2s K27me2 acetylated

K 7 * * H3 16-35 R26me2s K27me3 acetylated

**K 8 ** ** H3 16-35 R26me2s K27ac acetylated**

K 9 H3 16-35 R26me2s S28P acetylated

K10 * * H3 16-35 R26me2a K27me1 acetylated

K11 * * H3 16-35 R26me2a K27me2 acetylated

K12 * * H3 16-35 R26me2a K27me3 acetylated

**K13 ** * H3 16-35 R26me2a K27ac acetylated**

K14 H3 16-35 R26me2a S28P acetylated

K15 * * H3 16-35 R26Citr K27me1 acetylated

K16 * * H3 16-35 R26Citr K27me2 acetylated

K17 * * H3 16-35 R26Citr K27me3 acetylated

K18 H3 16-35 R26Citr S28P acetylated

K19 H3 16-35 K27me1 S28P acetylated

K20 H3 16-35 K27me2 S28P acetylated

K21 H3 16-35 K27me3 S28P acetylated

K22 H3 16-35 K27ac S28P acetylated

K23 H3 16-35 R26me2s K27me1 S28P acetylated

K24 H3 16-35 R26me2s K27me2 S28P acetylated

L 1 H3 16-35 R26me2s K27me3 S28P acetylated

L 2 H3 16-35 R26me2s K27ac S28P acetylated

L 3 H3 16-35 R26me2a K27me1 S28P acetylated

L 4 H3 16-35 R26me2a K27me2 S28P acetylated

L 5 H3 16-35 R26me2a K27me3 S28P acetylated

L 6 H3 16-35 R26me2a K27ac S28P acetylated

L 7 H3 26-45 unmod acetylated

L 8 H3 26-45 K36me1 acetylated

L 9 H3 26-45 K36me2 acetylated

L10 H3 26-45 K36me3 acetylated

L11 H3 26-45 K36ac acetylated

L12 H4 1-19 unmod free

L13 H4 1-19 S1P free

L14 H4 1-19 R3me2s free

L15 H4 1-19 R3me2a free

L16 H4 1-19 K5ac free

L17 H4 1-19 K8ac free

L18 H4 1-19 K12ac free

L19 H4 1-19 K16ac free

L20 H4 1-19 S1P R3me2s free

L21 H4 1-19 S1P R3me2a free

L22 H4 1-19 S1P K5ac free

L23 H4 1-19 R3me2s K5ac free

L24 H4 1-19 R3me2s K8ac free

M 1 H4 1-19 R3me2a K5ac free

M 2 H4 1-19 R3me2a K8ac free

M 3 H4 1-19 K5ac K8ac free

M 4 H4 1-19 K8ac K12ac free

M 5 H4 1-19 K8ac K16ac free

M 6 H4 1-19 K12ac K16ac free

M 7 H4 1-19 S1P R3me2s K5ac free

M 8 H4 1-19 S1P R3me2a K5ac free

M 9 H4 1-19 R3me2s K5ac K8ac free

M10 H4 1-19 R3me2a K5ac K8ac free

M11 H4 1-19 K5ac K8ac K12ac free

M12 H4 1-19 K8ac K12ac K16ac free

M13 H4 1-19 S1P R3me2s K5ac K8ac free

M14 H4 1-19 S1P R3me2a K5ac K8ac free

M15 H4 1-19 R3me2s K5ac K8ac K12ac free

M16 H4 1-19 R3me2a K5ac K8ac K12ac free

M17 H4 1-19 K5ac K8ac K12ac K16ac free

M18 H4 11-30 unmod acetylated

M19 H4 11-30 K12ac acetylated

M20 H4 11-30 K16ac acetylated

M21 H4 11-30 R17me2s acetylated

M22 H4 11-30 R17me2a acetylated

M23 H4 11-30 R19me2s acetylated

M24 H4 11-30 R19me2a acetylated

N 1 H4 11-30 K20me1 acetylated

N 2 H4 11-30 K20me2 acetylated

N 3 H4 11-30 K20me3 acetylated

N 4 H4 11-30 K20ac acetylated

N 5 H4 11-30 R24me2a acetylated

N 6 H4 11-30 R24me2s acetylated

N 7 * H4 11-30 K12ac K16ac acetylated

N 8 * H4 11-30 K16ac R17me2s acetylated

N 9 * H4 11-30 K16ac R17me2a acetylated

N10 H4 11-30 K16ac R19me2s acetylated

N11 * H4 11-30 K16ac R19me2a acetylated

N12 H4 11-30 K16ac K20me1 acetylated

N13 H4 11-30 K16ac K20me2 acetylated

N14 H4 11-30 K16ac K20me3 acetylated

N15 * H4 11-30 K16ac K20ac acetylated

N16 H4 11-30 K12ac K16ac K20me1 acetylated

N17 * H4 11-30 K12ac K16ac K20me2 acetylated

N18 H4 11-30 K12ac K16ac K20me3 acetylated

N19 * * H4 11-30 K12ac K16ac K20ac acetylated

N20 H4 11-30 R19me2a K20me1 acetylated

N21 H4 11-30 R19me2a K20me2 acetylated

N22 H4 11-30 R19me2a K20me3 acetylated

N23 * H4 11-30 R19me2a K20ac acetylated

N24 H4 11-30 R19me2s K20me1 acetylated

O 1 H4 11-30 R19me2s K20me2 acetylated

O 2 H4 11-30 R19me2s K20me3 acetylated

O 3 * H4 11-30 R19me2s K20ac acetylated

O 4 H4 11-30 R24me2a K20me1 acetylated

O 5 H4 11-30 R24me2a K20me2 acetylated

O 6 H4 11-30 R24me2a K20me3 acetylated

O 7 H4 11-30 R24me2a K20ac acetylated

O 8 H4 11-30 R24me2s K20me1 acetylated

O 9 H4 11-30 R24me2s K20me2 acetylated

O10 H4 11-30 R24me2s K20me3 acetylated

O11 H4 11-30 R24me2s K20ac acetylated

O12 H2a 1-19 unmod free

O13 H2a 1-19 S1P free

O14 H2a 1-19 K5ac free

O15 H2a 1-19 K9ac free

O16 H2a 1-19 K13ac free

O17 H2a 1-19 S1P K5ac free

O18 H2a 1-19 S1P K9ac free

O19 H2a 1-19 S1P K13ac free

O20 H2a 1-19 K5ac K9ac free

O21 H2a 1-19 K5ac K13ac free

O22 H2a 1-19 K9ac K13ac free

O23 H2a 1-19 S1P K5ac K9ac free

O24 H2a 1-19 S1P K5ac K13ac free

P 1 H2a 1-19 S1P K9ac K13ac free

P 2 H2a 1-19 K5ac K9ac K13ac free

P 3 H2a 1-19 S1P K5ac K9ac K13ac free

P 4 H2B 1-19 unmod free

P 5 H2B 1-19 K5ac free

P 6 * H2B 1-19 K12ac free

P 7 H2B 1-19 S14P free

P 8 * H2B 1-19 K15ac free

P 9 H2B 1-19 K5ac K12ac free

P10 H2B 1-19 K5ac S14P free

P11 * H2B 1-19 K5ac K15ac free

P12 H2B 1-19 K12ac S14P free

P13 H2B 1-19 K12ac K15ac free

P14 * H2B 1-19 S14P K15ac free

P15 H2B 1-19 K5ac K12ac S14P free

P16 H2B 1-19 K5ac K12ac K15ac free

**P17 ** H2B 1-19 K5ac S14P K15ac free**

P18 * H2B 1-19 K12ac S14P K15ac free

P19 * H2B 1-19 K5ac K12ac S14P K15ac free

P20 Biotin, control peptide biotinylated

P21 c-myc tag free

P22 neg. control acetylated

P23 background 01 acetylated

P24 background 02 acetylated
